# The Flavonol Isoquercitrin Promotes Mitochondrial-Dependent Apoptosis in SK-Mel-2 Melanoma Cell via the PI3K/AKT/mTOR Pathway

**DOI:** 10.3390/nu12123683

**Published:** 2020-11-29

**Authors:** Yeong-Seon Won, Jeong-Ho Kim, Rona Camille M. Lizardo, Hye-Ji Min, Hyun-Dong Cho, Seong-Min Hong, Kwon-Il Seo

**Affiliations:** 1 Department of Food Biotechnology, Dong-A University, Busan 49315, Korea; wonys@dau.ac.kr (Y.-S.W.); rmlizardo@up.edu.ph (R.C.M.L.); alsgpwl1473@naver.com (H.-J.M.); 2 Department of Food Science and Technology, Kyungpook National University, Daegu 41566, Korea; kimjeoho90@gmail.com; 3 Institute of Food Science and Technology, College of Agriculture and Food Science, University of the Philippines Los Baños, College, Laguna 4031, Philippines; 4 Department of Pharmacology & Regenerative Medicine, University of Illinois, Chicago, IL 60612, USA; chd0811@hanmail.net; 5 College of Pharmacy and Gachon Institute of Pharmaceutical Science, Gachon University, Incheon 21936, Korea; hongsm0517@gmail.com

**Keywords:** isoquercitrin, flavonol, human melanoma, mitochondrial-mediated apoptosis, natural chemopreventive agent

## Abstract

Isoquercitrin (IQ), a major flavonol present in *Prunus mume* fruit, has gained much attention in recent studies because of its superior bioavailability and physiological effects. In this study, the anti-cancer mechanism of IQ against human melanoma, particularly its effect on the mitochondria-mediated apoptosis, was investigated. Treatment with IQ at 25 μM concentration effectively inhibited the proliferation of SK-MEL-2 skin cancer cells while the same concentration did not exhibit cytotoxicity against human keratinocytes HaCaT. Morphological analysis and clonogenic assay also showed that IQ can alter the growth and long-term survival of SK-MEL-2 cells. IQ also induced apoptosis in the melanoma cells as manifested in the nuclear morphology changes, DNA fragmentation, increase in the apoptosis rate (17.69% at 25 μM) and accumulation of sub-G1 cell cycle phase population (19.55% at 25 μM). Western blot analysis revealed the involvement of the mitochondrial apoptosis signaling pathway in the anti-cancer property of IQ. Treatment with IQ resulted in the decrease in the levels of procaspase-8 and -9, and Bcl-2 protein, and an increase in the expression of cleaved PARP and Bax. Moreover, AIF and Endo G protein expression increased, indicating a caspase-independent mitochondrial-mediated apoptosis. The anti-proliferative activity of IQ against SK-MEL-2 can also be attributed to the downregulation of the PI3K/AktmTOR signaling pathway. These findings showed that IQ can be developed into a chemopreventive therapeutic agent against the melanoma cells.

## 1. Introduction

Flavonoids are a diverse group of compounds which are ubiquitously distributed among plants, especially in fruits and vegetables. Being a regular and essential part of the human diet, their toxicity is considered as negligible. In addition, their biological activities, benefits to health, as well as pharmacological properties have been an important focus of several studies. Among the vast group of flavonoids, some of the most well-known compounds are the polyphenols under the subgroup flavonol, which include quercetin and its derivatives [1]. Isoquercitrin (IQ) or quercetin-3-O-*β*-D-glucopyranoside is a major glycosidic derivative of the naturally and abundantly occurring flavonoid quercetin, alongside rutin. Similar to other flavonols, IQ is often found in fruits, vegetables, cereals, juices and beverages, and other plant-based foods [2]. A considerable amount of IQ has been found and isolated from Prunus mume [3], also known as Japanese apricot, a traditional food and medicinal plant which originated from Southern part of China and was further cultivated in East Asian countries including Taiwan, Japan, and Korea [4]. Different extracts of Prunus mume fruit, including ethyl acetate extract [5] and methanol extract [6], contain IQ, to which the biological activities of these extracts can be largely attributed.

Previous studies about the biological activities, specifically the chemopreventive potential, have been intensively conducted about the flavonols quercitin and rutin. Conversely, only few studies have published data about the equally important and biologically active compound, isoquercitrin. In addition, IQ is more soluble in aqueous phase and has greater bioavailability as compared with the aglycone, quercetin, making it highly suitable in many food and pharmacological applications [2]. The biological activities of IQ were vastly influenced by its antioxidant and anti-inflammatory activities. In vitro studies conducted on IQ were mostly focused on its ROS scavenging activity, anti-angiogenesis, inhibition of melanogenesis, and anti-carcinogenic properties [2]. In a study performed by Li et al. (2016), it was discovered that IQ exhibited greater activity in terms of the Fe^2+^-binding, ET-based ferric ion reducing antioxidant power, and multi-pathways-based superoxide anion-scavenging as compared with the well-studied quercetin [7]. Meanwhile, the findings of Matsubara et al. (2004) showed that IQ exhibits high anti-angiogenesis effect, which is also an important manifestation of anti-cancer potential [8]. Studies about the anti-cancer properties, particularly breast, colon, liver, prostate, fibrosarcoma, pancreatic, and cerebral cancers, of isoquercitrin and its modified forms have been conducted in the past years [9] but no recent reports were published about the mechanisms behind the effect of IQ against the melanoma cells.

Melanoma, which is brought about by malignant changes in the pigment-producing cells, is considered as one of the most aggressive types of cancer [10,11]. The percentage of incidence of malignant melanoma has been rising recently at a relatively fast rate, accounting for approximately 1.6% new cancer cases on an annual basis [12]. This scenario is considered unusual considering the demographics of the occurrence of the disease such as the age and the region. The socio-economic burden of melanoma is construed to be unbalanced, and interestingly, the highest incidence was found among the younger population [10]. The most significant risk factors were environmental aspects, particularly prolonged exposure to UV radiation, as well as the individual genetics [10,13]. Although advances in the treatment of melanoma, even the metastatic and malignant forms, have been made available in the past years, the efficacy of these therapies are still being explored and identification of promising combination of treatments is still under study [14,15].

Apoptosis is a physiological phenomenon involving the programmed cell death and is an important process for the control of cell population and systematic removal of damaged cells with impaired DNA [16,17]. Deregulation in the normal path of apoptosis is one of the main causative factors in many diseases including cancer development and progression. In melanoma, most anti-cancer therapy and treatment approaches commonly targeted serine/threonine protein kinase inhibitors, particularly those involved in the Ras/Raf/MEK/ERK and PI3K/PTEN/Akt/mTOR signaling pathways [11].

The present study aimed to investigate the effect of a major flavonol found in *Prunus mume*, isoquercitrin, in the viability of melanoma and normal skin cells, and to elucidate the mechanism, particularly the apoptotic cell death-related signaling pathways, behind the anti-cancer effect of IQ.

## 2. Materials and Methods 

### 2.1. Chemicals and Reagents

**Figure 1:**
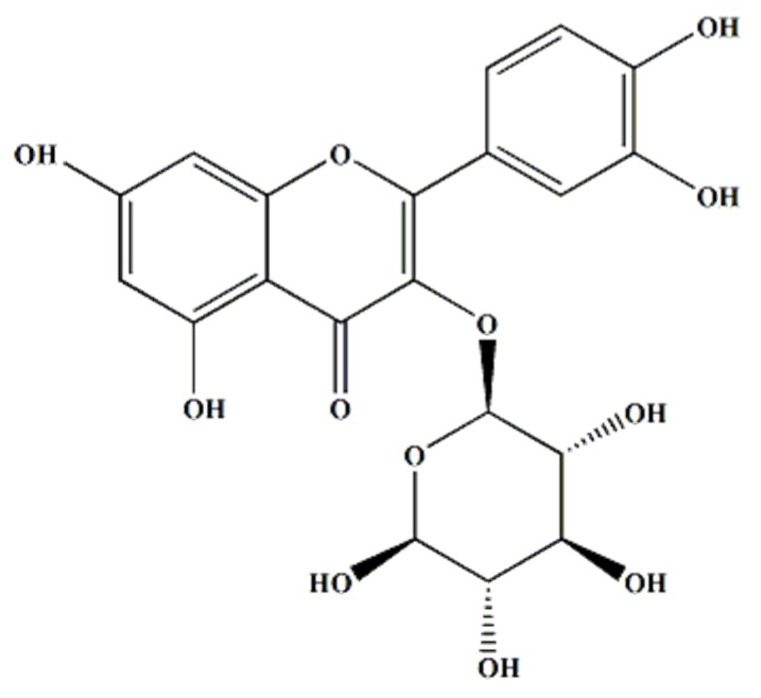
Chemical structure of isoquercitrin.

Isoquercitrin or quercetin-3-O-*β*-D-glucopyranoside (Figure 1) was obtained from Sigma-Aldrich (St. Louis, MO, USA). Dulbecco’s modified eagle medium (DMEM), RPMI 1640 medium, fetal bovine serum (FBS), trypsin-EDTA, penicillin, and antibiotic-antimycotic were purchased from GIBCO BRL Co. (Gaithersburg, MD, USA). Sulforhodamin B (SRB), RNase, and Hoechst 33258 bis-benzamide were purchased from Sigma-Aldrich Co. Ltd. (St. Louis, Missouri USA). Anti-Bid (sc-514622), anti-Bax (sc-7480), anti-Bcl-2 (sc-7382), anti-procaspase-3, anti-procaspase-8, anti-procaspase-9, anti- Endonuclease G (Endo G) (sc-365359), anti-AIF (sc-13116), anti-poly (ADPribose) polymerase-1 (PARP-1) (sc-56197), anti-Akt (sc-5298), anti-p-Akt (sc-7985-R), anti-mTOR (sc-8319), anti-p-mTOR (sc-101738), anti-PI3K (sc-423), and anti-β-actin (sc-47778) antibodies were procured from Santa Cruz Biotechnology (Santa Cruz, CA, USA). The ECL kit was purchased from Amersham Life Science (Amersham, UK).

### 2.2. Cell Culture

SK-MEL-2 (malignant melanoma cells derived from metastasis on human thigh skin), SK-MEL-28 (malignant melanoma cells derived from human skin), B16 (melanoma cells derived from mouse skin tissue), and HaCaT (normal keratinocytes derived from adult human skin) were purchased from American Type Culture Collection (ATCC, Rockville, ND, USA). The cells were cultured in RPMI (SK-MEL-2) and DMEM (SK-MEL-28, B16, and HaCaT) supplemented with 10% fetal bovine serum (FBS), penicillin and streptomycin (Gibco, ThermoScientific Co., Waltham, MA, USA). The cultures were incubated and maintained at humidified atmosphere condition with 5% CO2 at 37 °C.

### 2.3. Cell Proliferation Analysis via Sulforhodamin B (SRB) Assay

The anti-proliferative activity of isoquercitrin against both the normal skin and melanoma cell lines was evaluated using the sulforhodamin B assay. The normal skin cell HaCaT and the human melanoma cells, namely SK-MEL-28, SK-MEL-2, and murine melanoma B16, were seeded at a density of 3 × 104 cells/well in 48-well cell culture plates and treated with various concentrations of isoquercitrin. After incubation for 24, 48, and 72 h, the medium was removed and 10% trichloro-acetic acid was added. After 1 h of incubation at 4 °C, the wells were washed and the cells were stained with 0.4% (w/v) SRB at room temperature for 1 h and then washed using 1% acetic acid. Bound SRB was solubilized with 10 mM Tris and the absorbance was measured at 540 nm using a microplate reader (Molecular Devices, Inc., California, USA).

### 2.4. Clonogenic Survival Assay

The ability of HaCaT keratinocytes and SK-MEL-2 melanoma cells to form colonies after long-term treatment with isoquercitrin was evaluated. Briefly, 2 × 104 cells were seeded on a 6-well culture plate. After 24 h, the cells were treated with 25 μM of IQ for another 24 h and incubated at 37 °C. Fresh medium was replaced every after 24–48 h until the 14 days of incubation. Colony formation was evaluated and photographed after fixing the cells with 100% methanol and staining with 0.5% crystal violet. 

### 2.5. Evaluation of Morphological Apoptosis 

The morphological changes in the SK-MEL-2 cells undergoing apoptosis were analyzed using the Hoechst staining assay and detected by fluorescent microscopy. The cells were first seeded in 6-well plates at a density of 1 × 106 cells per well. Then, the cells were subjected to treatment with 15, 20, and 25 μM IQ for 24 h. For the staining assay, the cells were harvested, washed with PBS and stained with 200 μL of Hoechst 33258 bis-benzamide (5 μg/mL) for 10 min at room temperature. Then, cell suspension was placed on the glass slide and examined using a fluorescence microscope (Olympus Optical, Co., Ltd., Tokyo, Japan).

### 2.6. DNA Fragmentation Analysis

SK-MEL-2 cells were seeded on a 100-mm dish at a density of 2 × 106 and cultured in RPMI medium for 24 h. Then, the cells were treated with the indicated concentrations of IQ for 24 h. After treatment, the cell suspensions were centrifuged and the cells were lysed using the lysis buffer consisting of 10 mM Tris-HCl, 10 mM EDTA, 0.5% Triton X-100, 20% SDS, and 10 mg/mL proteinase K. Then, extraction was performed with phenol:chloroform:isoamyl alcohol (25:24:1) solution and DNA was precipitated with cold absolute ethanol. The resulting pellets were incubated in Tris-HCl EDTA buffer and RNAse (2 mg/mL) for 1 h at 37 °C. Electrophoresis separation was performed on 2% agarose gel containing ethidium bromide. The resulting DNA bands were visualized and examined using the UV Trans illuminator imaging system (Entela Co. Ltd., Entzheim, France).

### 2.7. Apoptotic Cell Death Analysis Using Annexin V/PI Staining Assay

Analysis of the apoptotic cell death was performed using the Muse^®^ Annexin V/PI and dead cell assay kit according to the manufacturer’s protocol (Merck KGaA, Darmstadt, Germany). SK-MEL-2 cells were seeded into 24-well plates at 1 × 105 cells/well density and incubated for 24 h. The indicated concentrations of IQ were treated into the cells for another 24 h. Then, the cells were trypsinized, washed with PBS, and added with the staining reagent. Suspensions of the stained cells were transferred into microcentrifuge tubes and incubated for 20 min at room temperature. Flow cytometry, obtaining data from 5000 events (gated cells) per sample, was performed using Muse cell analyzer (Merck KGaA, Darmstadt, Germany). The percentages of cells were calculated from the mean fluorescence intensity in each of the four quadrants. 

### 2.8. Sub-G1 Population Analysis

Flow cytometry was performed to quantify the sub-G1 population in the cell cycle phase. SK-MEL-2 melanoma cells were seeded at a density of 1 × 106 cells/well in 6-well plates and cultured in RPMI for 24 h. The cells were then collected and fixed in ice-cold 70% ethanol solution and stored at 4 °C overnight. The cells were then washed and incubated in a medium containing 1 μL of 1 mg/mL RNAse, 20 μL of 1 mg/mL propidium iodide and 500 mL PBS at 37 °C for 30 min. Flow cytometry (BD FACS, CantoTM II Flow cytometry, BD Biosciences, San Jose, USA) was used to analyze the sub-G1 DNA content.

### 2.9. Western Blot Analysis

SK-MEL-2 melanoma cells were seeded at a density of 5 × 106 cells in a 100-mm cell culture plate and cultured for 24 h in RPMI medium. Then, the cells were treated with 25 μM of isoquercitrin for 24 h. After the treatment time, the cell suspensions were centrifuged and the resulting pellets were lysed in a medium containing the lysis buffer (50 mM Tris-HCl, 150 mM NaCl, 1 mM EDTA, 50 nM NaF, 30 mM Na4P2O7, 1 mM PMSF, and 2 μg/mL aprotinin) for 30 min on ice. The cell lysates were obtained and the total protein content in the supernatant was measured using a Bicinchoninic acid(BCA) protein kit (Pierce, Rockford, IL, USA). The extracted protein was then subjected to separation by SDS-PAGE. Protein detection was performed using the ECL kit (Santa Cruz, CA, USA).

### 2.10. Statistical Analysis

All data were performed in triplicate and the data obtained were subjected to one-way analysis of variance followed by Dunnett’s test or unpaired Student’s *t*-test. Results are expressed as a percentage. Data values are expressed as mean ± SD of triplicate determinations. Significant difference was established using Dunnett’s test or unpaired Student’s *t*-test at * *p* < 0.05, ** *p* < 0.01, and *** *p* < 0.001. Statistical analysis was conducted using the Prism software (GraphPad, La Jolla, CA, USA).

## 3. Results

### 3.1. Effects of Isoquercitrin on the Proliferation of Skin Cancer Cells

**Figure 2:**
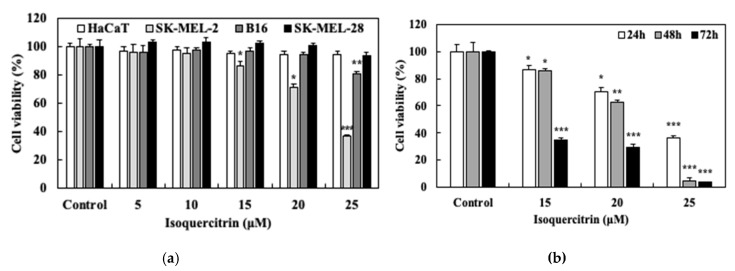
Effect of isoquercitrin on the viability of melanoma cells. (**a**) HaCaT normal skin cells, SK-MEL-2 and SK-MEL-28 human skin cancer cells, and B16 murine melanoma cells were treated with 5, 10, 15, 20, and 25 μM of isoquercitrin for 24 h. SRB assay was performed to measure the cell viability. (**b**) SK-MEL-2 melanoma cells were treated with 15, 20, and 25 μM of isoquercitrin for 24, 48, and 72 h. SRB assay was performed to evaluate the cell viability. Results are expressed as a percentage. Data values are expressed as mean ± SD of triplicate determinations. Significant difference was established using Dunnett’s test at * *P* < 0.05, ** *P* < 0.01 and *** *P* < 0.001.

To evaluate the effect of isoquercitrin (IQ) on the growth and proliferation of normal skin cell line HaCat and skin cancer cell lines SK-Mel-2, B16, and SK-Mel-28, the cells were cultured and treated with 5, 10, 15, 20, and 25 μM of IQ for 24 h. After performing the SRB assay, the results showed that isoquercitrin significantly inhibited the proliferation of SK-MEL-2 starting at a concentration of 15 μM (Figure 2a). Significant reduction in the cell viability down to 36.56% (*P* < 0.001) was reached by treatment with 25 μM of IQ. In contrast, the proliferation of SK-MEL-2 human skin cancer cells and B16 murine melanoma cells were not suppressed by the increasing concentrations of IQ. Moreover, the compound did not affect the viability of the HaCaT human keratinocytes. 

SK-MEL-2 cell was employed in further experiments in order to establish the effect of IQ on its viability. The cells were further subjected to treatment with 15, 20, and 25 μM of IQ for 24, 48, and 72 h. As shown in Figure 2b, the results indicated that treatment with IQ at an increasing exposure time caused the reduction in SK-MEL-2 proliferation in a dose-dependent manner. Likewise, the highest inhibition rate was achieved through treatment with IQ for 72 h, reducing the viability of the cells by more than 60% starting at the 15 μM concentration. These results demonstrated that isoquercitrin can significantly inhibit the proliferation of melanoma cells, specifically SK-MEL-2, in a time- and dose-dependent manner without exhibiting cytotoxicity against normal skin cells.

### 3.2. Isoquercitrin Inhibits Cell Growth and Clonogenic Survival of SK-MEL-2

**Figure 3:**
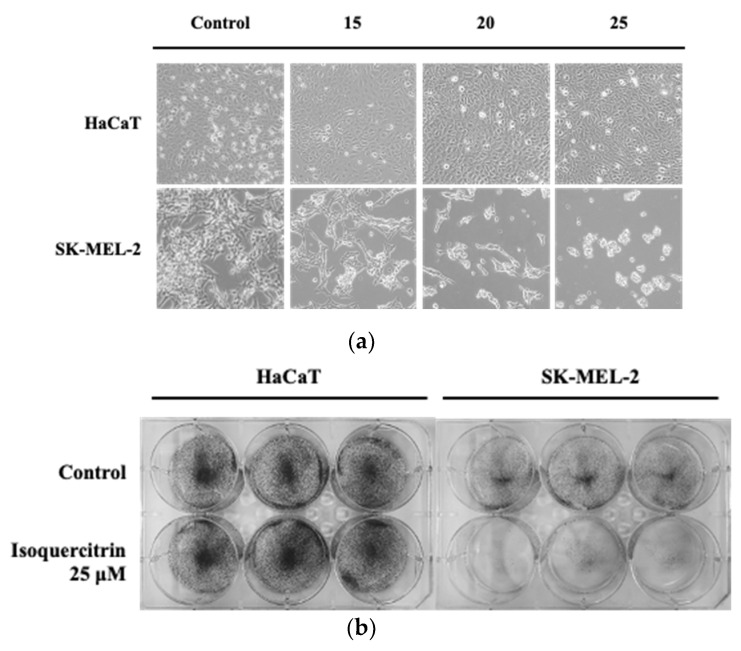
Effect of isoquercitrin on the growth morphology and clonogenicity of SK-MEL-2 cells. HaCaT normal skin cells and SK-MEL-2 melanoma cells were treated with 15, 20, and 25 μM of isoquercitrin. (**a**) After 24 h incubation, the morphology of cells was visualized and compared using an inverted microscope (magnification, 200×). (**b**) Clonogenic assay was performed after incubation for 14 days at 37 °C.

The effect of treatment with isoquercitrin on the morphology and growth of SK-MEL-2 was assessed and compared with that of the normal skin HaCaT cells. As illustrated in the photomicrographs (Figure 3a), IQ had no pronounced effect on the growth and morphology of HaCaT cells. Meanwhile, it is evident that treatment with increasing concentrations of IQ altered the morphological characteristics and confluency of SK-MEL-2 cells. Under normal growth condition, the SK-MEL-2 cells appeared to have a polygonal shape with elongated dendritic morphology. Conversely, morphological changes in the melanoma cells treated with isoquercitrin were observed as the cells exhibited a slightly round shape and shrunken appearance. Furthermore, the cell growth and confluency were greatly reduced. 

Results of the clonogenic survival assay also showed that treatment with 25 μM of IQ can inhibit the long-term proliferation and clonogenicity of SK-MEL-2 without affecting the normal human skin cells (Figure 3b). Taken together, these results showed that treatment with IQ can significantly alter the morphology and prevent the growth and long-term clonogenic survival of SK-MEL-2 melanoma cells. Moreover, IQ did not affect the morphology, growth and survival of HaCaT keratinocytes. 

### 3.3. Isoquercitrin Promotes Apoptotic Cell Death in SK-MEL-2 Cells

**Figure 4:**
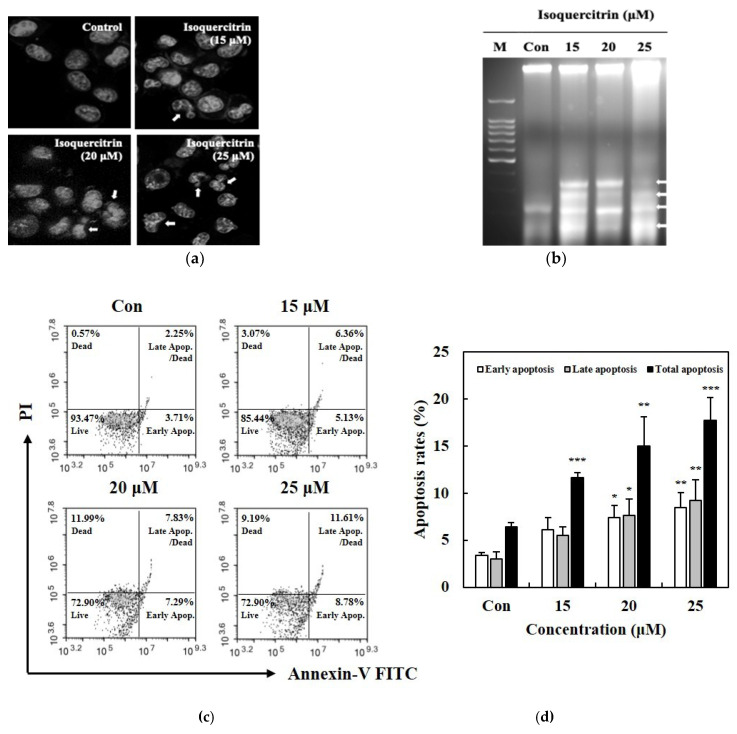
Effect of isoquercitrin on the apoptotic cell death of SK-MEL-2. The SK-MEL-2 cells were subjected to treatment with 15, 20, and 25 μM of isoquercitrin. (**a**) Nuclear condensation and membrane blebbing (indicated by the arrows) as affected by the treatment with isoquercitrin was observed using the Hoechst assay. (**b**) DNA fragmentation was detected through agarose gel electrophoresis. (**c**) Apoptotic cell death was analyzed using the Annexin V/PI staining assay. (**d**) The percentage of apoptotic cells was quantified. Results are expressed as a percentage. Data values are expressed as mean ± SD of triplicate determinations. Significant difference was established using Dunnett’s test at * *P* < 0.05, ** *P* < 0.01 and *** *P* < 0.001.

In order to determine whether the anti-proliferative activity of isoquercitrin against SK-MEL-2 melanoma cells involves apoptotic cell death, we examined the nuclear morphology through the Hoechst staining assay, DNA fragmentation through the agarose gel electrophoresis method, and the number of cells undergoing apoptosis via the Annexin V/PI staining technique. In Figure 4a, cells treated with IQ displayed membrane blebbing and nuclear condensation, which are considered as manifestations of apoptosis. Likewise, as depicted in Figure 4b, IQ induced DNA fragmentation in SK-MEL-2 skin cancer cells. An increase in the percentage of cells in both the early and late apoptosis stages was also brought about by treatment with IQ in a concentration-dependent manner (Figure 4c). The total number of cells undergoing apoptotic cell death increased in a dose-dependent manner and reached about 17.69% after treatment with 25 μM of IQ (Figure 4d).

**Figure 5:**
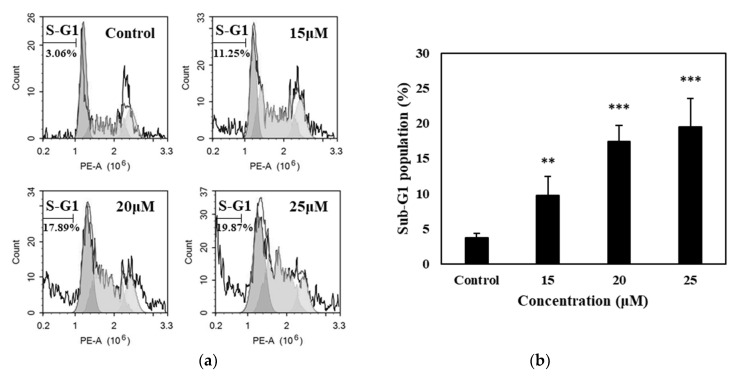
Isoquercitrin increases the sub-G1 population of SK-MEL-2 cells. (**a**) SK-MEL-2 cells were cultured in RPMI medium for 24 h and treated with the various concentrations of isoquercitrin. Sub-G1 population was determined through flow cytometry. (**b**) The Sub-G1 population was quantified. Results are expressed as a percentage. Data values are expressed as mean ± SD of triplicate determinations. Significant difference was established using Dunnett’s test at ** *P* < 0.01 and *** *P* < 0.001.

Accumulation of cells in the sub-G1 cell cycle phase as a result of apoptotic cell death was also analyzed through flow cytometry. Treatment with IQ resulted in the increase in the cell population in the sub-G1 phase in a dose-dependent manner as illustrated in Figure 5. Particularly, an increase to 19.87% in the number of cells that accumulated in the sub-G1 phase was detected upon treatment with 25 μM of IQ. These morphological and physiological changes in the cells manifested in the results of the experiments showed that IQ can induce apoptotic cell death in SK-MEL-2 melanoma cells.

### 3.4. Isoquercitrin Induces Apoptosis in SK-MEL-2 Cells through the Mitochondrial Apoptosis Pathway 

**Figure 6:**
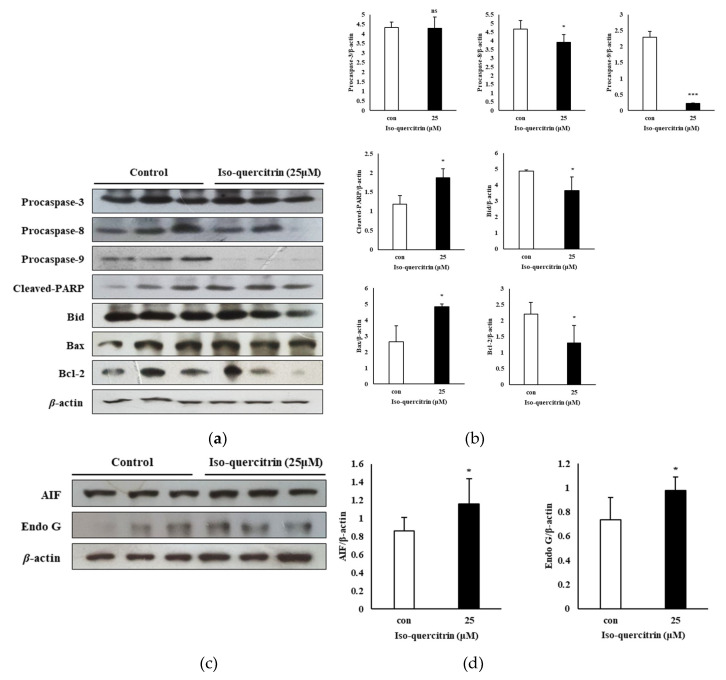
Isoquercitrin induces mitochondrial-mediated apoptosis in SK-MEL-2 cells. SK-MEL-2 cells were cultured in RPMI medium for 24 h and treated with 25 μM of isoquercitrin. Effect of IQ on the expression of (**a**) procaspase-3, -8, and -9, cleaved PARP, Bid, Bax, and Bcl-2; and (**c**) AIF and Endo G in SK-MEL-2 cells was analyzed through the western blot technique. (**b**,**d**) Total apoptotic proteins were quantified using the ImageJ software program. Results are expressed as a percentage. Data values are expressed as mean ± SD of triplicate determinations. Significant difference was established using unpaired Student’s *t*-test at * *P* < 0.05 and *** *P* < 0.001.

In order to elucidate the mechanism behind the apoptotic cell death effect of IQ, SK-MEL-2 cells were subjected to treatment with 25 μM of IQ and the expression of the apoptosis-related proteins was analyzed. As depicted in Figure 6a,b, treatment with IQ brought about the downregulation of the anti-apoptotic protein Bcl-2 and a significant increase in the pro-apoptotic protein Bax (2.65 to 4.84) and cleaved-PARP (1.19 to 1.87), despite a decrease in the level of another pro-apoptotic protein Bid (4.86 to 3.66). The expression of the procaspases-8 (4.68) and -9 (2.29) were also downregulated to 3.90 and 0.23, respectively, although IQ treatment did not significantly affect the expression of procaspase-3. Meanwhile, the levels of expression of AIF and Endo G were elevated through treatment with IQ from 0.86 and 0.74 to 1.16 and 0.98, respectively (Figure 6c,d). These findings suggested that the IQ-induced apoptotic cell death can be attributed to the mitochondrial-related apoptotic pathway, involving both the caspase-dependent and independent mechanisms.

### 3.5. Isoquercitrin Suppresses the Viability of SK-MEL-2 by Downregulating the PI3K/AKT/mTOR Pathway

**Figure 7:**
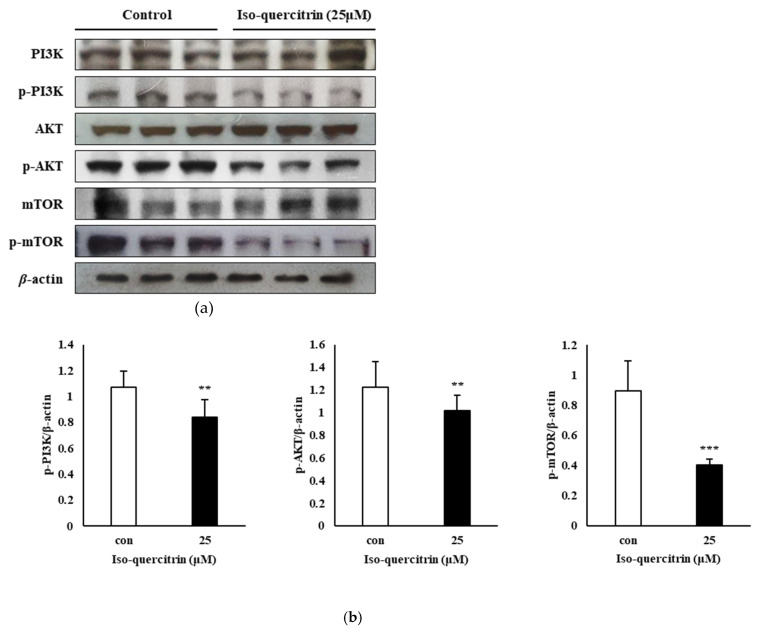
Isoquercitrin downregulates the PI3K/AKT/mTOR signaling pathway. (**a**) SK-MEL-2 cells were cultured in RPMI medium for 24 h and treated with 25 μM of isoquercitrin. Effect of IQ on the expression of phosphorylated PI3K, AKT, and mTOR in SK-MEL-2 cells was evaluated through the western blot analysis. (**b**) Results were quantified using the ImageJ software program. Results are expressed as a percentage. Data values are expressed as mean ± SD of triplicate determinations. Significant difference was established using unpaired Student’s *t*-test at ** *P* < 0.01 and *** *P* < 0.001.

The PI3K/AKT/mTOR signal transduction pathway is a known regulator of numerous cellular functions and events such as cell growth, survival and proliferation, as well as cell cycle progression and programmed cell death (Chang et al., 2003). To investigate the involvement of the PI3K/AKT/mTOR pathway on the inhibition of cell proliferation and initiation of apoptotic cell death induced by IQ, the effect of treatment with 25 μM of IQ was analyzed through the western blot technique. Figure 7 shows that treatment with IQ downregulated the expression of the phosphorylated PI3K (1.07 to 0.84), AKT (1.23 to 1.02) and mTOR (0.90 to 0.40) proteins. These results indicated that the IQ-induced SK-MEL-2 growth inhibition and cell death was mediated by the suppression of the PI3K/AKT/mTOR signaling pathway.

## 4. Discussion

*Prunus mume*, Japanese apricot, has been found to contain the flavonol compounds among the fruits in the Prunus genus. Furthermore, it contains an appreciable amount of 4.9 mg of the specific flavonol isoquercitrin per 100 g dry weight of fruit [3]. Isoquercitrin (IQ), a glycosylated derivative of the flavonoid quercetin, has gained attention as a focus for research in the past years because of its relatively higher specific biological activities, namely, its antioxidant property in terms of free-radical scavenging activity [7], its anti-angiogenic activity [8], as well as its anti-cancer property [9]. Plant extracts containing IQ as well as the sole compound isolated from different plant extracts revealed its great potential for treatment of various types of cancers such as pancreatic, colon, liver, breast and glioblastoma cancers. However, the mechanism behind the anti-tumor effect of IQ, especially in malignant melanoma, still remains ambiguous. In this study, we demonstrated that IQ promotes the mitochondrial apoptotic cell death of melanoma cells, particularly the SK-MEL-2, through the modulation of the PI3K/AKT/mTOR signaling pathway, without exhibiting cytotoxicity against normal skin cells.

Studies about the chemopreventive effect of IQ showed that this compound exhibits a wide spectrum of anti-proliferative activity against different types of cancer [9]. IQ displayed strong anti-proliferative activity against pancreatic cancer both in vitro and in vivo through induction of apoptosis and cell cycle arrest in pancreatic cancer cells [18]. Isoquercitrin isolated from *Hyptis fasciculata* also inhibited the proliferation of glioblastoma cell in a dose- and time-dependent manner [19]. Similarly, IQ reduced the growth and viability of three selected colon cancer cell lines, but did not affect the non-tumoral cell line tested [20]. In our experiments on the anti-proliferative activities of IQ, its treatment displayed potency against skin cancer cells, particularly the SK-MEL-2, by preventing the growth as well as the long-term survival of the tested melanoma cell without affecting the viability of the normal HaCaT keratinocytes. These findings were also in accordance with the results of previous studies on the effect of IQ on melanoma cells. De Freitas et al. (2016) showed that the ethanolic extract of *Morus nigra* L. leaves, containing IQ as one of the main flavonoids, exhibited higher cytotoxicity against murine carcinoma cell line [21]. Likewise, in the study performed by Ohguchi et al. (2010), IQ isolated from the peels of Japanese persimmon displayed an ability to inhibit melanogenesis in murine melanoma B16 [22].

Apoptosis has played a crucial role in the development and progression of cancer; hence, it has always become an important target for cancer treatments or therapies. In the present study, we have established in the results that the treatment with IQ can lead to apoptosis in SK-MEL-2 melanoma cells as manifested in the morphological changes, DNA fragmentation, and increase in the population of cells undergoing stages of apoptosis. Accumulation of the cells in the sub-G1 phase of the cell cycle was also evident, where DNA flocculation or partial relaxation of DNA occurs, and the cells lost some of their DNA on late stage of apoptosis.

Caspases play a fundamental role in apoptosis as these molecules serve as the initiator and executioner of the cell death process [23]. Isoquercitrin is one of the flavonoids which can target caspases [1]. In the current study, although a significant effect in the executioner caspase-3 was not evident in the results, treatment with IQ resulted in the downregulation of the expression of procaspase-8 and-9. This action leads to self-processing and activation of initiator caspases, which promotes the commencement of the apoptosis pathway [23,24]. Activation of related caspases also leads to the cleavage of the cellular protein poly(ADP-ribose) polymerase-1 (PARP-1), which further results in the destruction of apoptotic cells [25]. PARP is considered to be a pro-death signaling molecule as it serves as a nuclear and mitochondrial signal to release apoptosis inducing factor (AIF) from the mitochondria in PARP-1 dependent cell death [26]. The results of the present study showed the ability of IQ to increase the levels of cleaved PARP in SK-MEL-2 cells. Moreover, treatment with IQ brough about significant upregulation of AIF and Endo G. These signaling molecules are apoptosis promoting proteins which are released during the mitochondrial-associated pathway and can act independently of the caspase activation [27,28].

The Bcl-2 family of proteins are key regulators of the intrinsic pathway of apoptosis or the mitochondrial-associated pathway in cancer because the anti-apoptotic factors belonging to this family of proteins are often overexpressed in various types of cancer and are also crucial in the ability of tumor cells to evade apoptosis [29]. These proteins also primarily play a significant role in the regulation of the release of signaling molecules from the mitochondria which act as caspase activators [30]. This family of proteins is comprised both of pro-apoptotic and anti-apoptotic effectors, serving as an apoptotic cell death “switch” [17,29]. Consequently, the Bcl-2 family is an important target for therapeutic anti-cancer agents mainly by suppressing the activity of the anti-apoptotic proteins [23]. In the findings presented in this study, IQ treatment significantly upregulated the expression of the pro-apoptotic protein Bax while downregulating Bcl-2 expression. Taken together, the results showed that IQ brought about mitochondrial dysfunction and prompted apoptotic cell death in SK-MEL-2 melanoma cells by regulating signaling proteins associated with the mitochondrial apoptosis.

In the present study, we have also shown the involvement of the PI3K/AKT/mTOR signaling pathway in the mechanism behind the ability of IQ to inhibit the proliferation and survival of SK-MEL-2 melanoma cells. The PI3K/AKT pathway has a significant function in the cell survival mechanism and in promoting mitochondrial-associated apoptosis through its interaction with the Bcl-2 family protein, Bax. In the research conducted by Tsuruta, Masuyama, and Gotoh (2002), the PI3K/AKT pathway can block the Bax protein, leading to cell survival [31]. Hence, suppression of the PI3K/AKT pathway plays a role in the regulation of the mitochondrial pathway leading to apoptosis. The results of this study showed that IQ exhibits an anti-proliferative property against SK-MEL-2 human skin cancer cells by downregulating the expression of phosphorylated PI3K, AKT and mTOR signaling proteins.

## 5. Conclusions

The current study presented the mechanism behind the inhibition activity of isoquercitrin against human melanoma cells. IQ inhibited the proliferation and clonogenic survival of SK-MEL-2 skin cancer cells. Morphological examination and cell analysis through flow cytometry also revealed that IQ can induce apoptosis in SK-MEL-2 cells. The results of this study also indicate that IQ can inhibit the survival and can promote the mitochondrial-mediated apoptosis of SK-MEL-2 melanoma cells through the activation of both the caspase-dependent and -independent pathways and downregulation of the PI3K/AKT signaling pathway. The potential inhibitory effect of IQ using higher concentrations on other melanoma cells is recommended in further studies. In vivo studies may also be conducted to confirm the results that were established through the present study. Taken together, these findings can expand our understanding about IQ, suggesting its application in the development of a chemopreventive agent against the melanoma targeting the PI3K/AKT and mitochondrial-mediated apoptosis pathway.
